# Development of a 5G-Connected Ultra-Wideband Radar Platform for Traffic Monitoring in a Campus Environment

**DOI:** 10.3390/s25103203

**Published:** 2025-05-20

**Authors:** David Martín-Sacristán, Carlos Ravelo, Pablo Trelis, Miriam Ortiz, Manuel Fuentes

**Affiliations:** 1 iTEAM Research Institute, Universitat Politècnica de València, Camino de Vera s/n, Building 8G, D Access, 46022 Valencia, Spain; 2 5G Communications for Future Industry Verticals S.L. (Fivecomm), Camino de Vera s/n, Building 6D, 46022 Valencia, Spain; carlos.ravelo@fivecomm.eu (C.R.); pablo.trelis@fivecomm.eu (P.T.); miriam.ortiz@fivecomm.eu (M.O.); manuel.fuentes@fivecomm.eu (M.F.)

**Keywords:** traffic monitoring, UWB, radar, 5G, IoT platform

## Abstract

This paper presents the design, implementation, and testing of a traffic monitoring platform based on 5G-connected Ultra-Wideband (UWB) radars deployed on a university campus. The development of both connected radars and an IoT platform is detailed. The connected radars integrate commercial components, including a Raspberry Pi (RPi), a UWB radar, a standard enclosure, and a custom communication board featuring a 5G module. The IoT platform, which receives data from the radars via MQTT, is scalable, easily deployable, and supports radar management, data visualization, and external data access via an API. The solution was deployed and tested on campus, demonstrating real-time operation over a commercial 5G network with an estimated median latency between the radar and server of 75 ms. A preliminary evaluation conducted on a single radar during peak-hour traffic on a double-lane road, representing a challenging scenario, indicated a high detection rate of 94.81%, a low false detection rate of 1.02%, a high classification accuracy of 97.29%, and a high direction accuracy of 99.66%. These results validate the system’s capability to deliver accurate traffic monitoring.

## 1. Introduction

Effective traffic management is essential to addressing multiple urban challenges. Population growth and rapid urbanization have led to increased congestion and infrastructure strain, making efficient mobility systems a necessity [1]. At the same time, sustainability concerns have driven efforts to reduce environmental impact by optimizing transportation networks and minimizing emissions. Another key objective is enhancing mobility efficiency, ensuring smooth traffic flow, and achieving better integration with public transportation. Additionally, urban planners are focusing on creating safer, more livable cities by repurposing streets for pedestrian use, improving public transit, and implementing dynamic traffic control strategies.

Traffic monitoring plays a crucial role in enabling effective traffic management. It provides essential data on vehicle flow, congestion patterns, and mobility trends, forming the basis for informed urban planning decisions. Without monitoring, cities would lack the insights needed to optimize traffic flow, enhance public transportation, or implement sustainability measures. Furthermore, integrating traffic monitoring with smart city infrastructure enables predictive analytics, allowing authorities to proactively address congestion and safety risks [2,3].

Traditional traffic monitoring systems, including optical cameras, inductive loop sensors, and other in situ technologies, face well-documented limitations in installation complexity, operational reliability, and adaptability to varying environments [4,5,6,7,8,9]. Intrusive technologies, such as inductive loops and embedded magnetometers, require physical installation within or beneath the pavement surface, leading to disruptive civil works, traffic lane closures, and long-term degradation of road surfaces [5]. Their installation and maintenance increase costs and can compromise road safety during deployment. These systems are also susceptible to mechanical wear from repeated vehicle stresses and thermal cycling, and are difficult to reposition or reconfigure once deployed.

Inductive loops are recognized for their accuracy in basic traffic metrics such as vehicle counts or occupancy. Accuracy ranging from 92% up to 99% is reported in multiple references in [8]. However, their performance often degrades when used in environments with diverse vehicle classes or high traffic dynamics [4]. Magnetometers and magnetic sensors offer installation flexibility (e.g., under bridges) but suffer from limited detection zones, necessitating multiple units for full lane coverage. Moreover, many magnetic systems struggle to detect stopped or slow-moving vehicles, which limits their utility in congested urban settings or for real-time actuation of traffic signals.

Non-intrusive systems, such as video image processors and radar detectors, reduce installation complexity but introduce other challenges. Video-based systems provide classification accuracy over 95% [8]. However, they depend heavily on favorable lighting and visibility conditions; their performance deteriorates significantly in low light, very high light, rain, fog, or snow. They also require regular lens cleaning and recalibration, making them maintenance-intensive. Additionally, video monitoring raises serious privacy concerns due to the potential capture of identifiable information, such as vehicle plates or pedestrian faces. High-resolution video streams generate large volumes of data, increasing the strain on network infrastructure and requiring substantial computing power for real-time analysis and storage. These systems also demand considerable mounting heights (9–15 m) and are vulnerable to vibration, occlusion, and shadows, which can impair detection accuracy.

Other non-visual sensors, such as ultrasonic, passive infrared, microwave, and acoustic detectors, may be sensitive to temperature shifts, environmental noise, or specific vehicle behaviors (e.g., stop-and-go traffic), limiting their general applicability. Some technologies, such as continuous-wave Doppler radar, cannot detect stationary vehicles. Laser radar and active infrared systems may be more robust but are also susceptible to visibility issues in fog or heavy snow, and their cost and maintenance demands remain high due to lens cleaning and alignment.

Ultra-Wideband (UWB) radar technology [10,11] emerges as a promising alternative among non-visual sensing methods, offering non-intrusive, weather-resilient, and privacy-preserving traffic monitoring capabilities. UWB radars can be mounted above or adjacent to the road surface, eliminating the need for pavement cuts and enabling relatively fast and flexible deployment. They maintain stable detection performance across adverse environmental conditions (such as fog, rain, and low-light scenarios) where optical and infrared systems typically degrade. Moreover, unlike video-based systems, UWB radars do not generate visual data, significantly reducing privacy risks and alleviating the demand for high-bandwidth data transmission and intensive image processing.

However, like other non-visual sensors, UWB radars also inherit some limitations that must be carefully considered. These systems often face challenges in accurately detecting slow-moving or stationary vehicles, particularly in congested or stop-and-go traffic conditions, unless specific signal processing algorithms or advanced sensor layouts are implemented [4]. Their performance can also be influenced by the radar’s angle of installation, mounting height, and lane width, which require careful calibration to ensure optimal coverage and accuracy. Furthermore, while less affected by weather than cameras, radar systems may still experience multipath reflections or interference in complex urban environments, such as near tall buildings or metallic surfaces. In multi-lane scenarios, detection accuracy may decrease due to cross-lane interference or insufficient angular resolution, potentially requiring multiple sensors for full scene coverage. Lastly, while installation is more straightforward than in-road sensors, practical deployment still requires secure mounting infrastructure, power sources, and reliable data communication channels, especially when integrated into IoT platforms.

While UWB radar systems offer a compelling balance of privacy, environmental resilience, and non-intrusive deployment, their practical performance in real-world traffic scenarios depends significantly on system design choices, including radar frequency, sensor integration, and platform connectivity. Recent studies have explored a range of UWB radar-based traffic monitoring solutions, each addressing different aspects of the sensing pipeline, from raw signal acquisition to classification and system deployment. However, many of these works fail to offer an integrated, end-to-end solution that is practically deployable and scalable within a modern smart city infrastructure.

In [11], the focus is on the design of a 24.1 GHz radar system, detailing both hardware components (such as antenna systems and transceivers) and associated software. A similar approach is presented in [12], which explores the design of a Frequency-Modulated Continuous-Wave (FMCW) radar operating at 35 GHz. However, these works do not emphasize detection or classification performance and do not include results from real-world measurements. In [13], an FMCW radar system based on the Texas Instruments IWR1642 module (operating at 77 GHz) is presented. This study includes real-world measurements in various urban scenarios, achieving up to 95% classification accuracy in a six-lane bidirectional traffic environment, characterized by relatively smooth traffic flow with few stops or congestion. The accuracy drops to approximately 90% in more complex urban scenarios. However, this work does not integrate the radar with an IoT platform. In contrast to our system, these previous works either do not utilize commercial off-the-shelf (COTS) components or do not combine them into a deployable, compact form factor.

The work in [6] presents a portable radar system using the commercial X4M03 radar module and an ESP32 microcontroller with wireless connectivity (Wi-Fi, Bluetooth). This system achieves detection rates above 98% and classification accuracies exceeding 96% in the case of multi-lane, single-direction roads under varying speeds. However, it lacks integration with a broader IoT platform and does not support cellular connectivity, which limits scalability and remote access capabilities.

The advantages of UWB radar systems are further enhanced when combined with 5G connectivity. Fifth-generation networks enable low-latency, high-bandwidth transmission of sensor data, supporting real-time system management and the integration of distributed sensing units into scalable traffic monitoring networks [14,15]. Unlike video-based systems, the data footprint of radar signals is significantly lower, reducing communication and processing overhead. Furthermore, the remote manageability offered by 5G allows for system reconfiguration, monitoring, and troubleshooting without on-site intervention, enhancing economic feasibility and operational resilience. Together, the fusion of UWB radar and 5G communication represents an innovative direction in intelligent transportation systems, addressing many of the critical shortcomings observed in traditional and contemporary monitoring technologies, particularly in terms of installation complexity, cost, environmental adaptability, maintenance, data efficiency, and privacy.

While prior research has explored the use of radar- and Internet of Things (IoT)-based traffic monitoring, there remains a gap in understanding the practical issues of the integration of UWB radar with Fifth-Generation (5G) networks in real-world deployments. Our work focuses on developing a complete end-to-end readily deployable system using easily assembled COTS hardware. Unlike prior studies, we integrate radar sensing with a cellular-connected IoT platform (leveraging commercial 4G/5G networks), enabling low-latency data transmission, remote system management, and broad-area coverage. We also validate detection and classification performance through empirical testing in a real-world environment. In summary, this paper presents the design, implementation, and testing of a 5G-connected UWB radar platform for urban traffic monitoring, deployed on the Vera campus of the Universitat Politècnica de València (UPV).

The primary contributions of this work are as follows:

- Development of a scalable and deployable 5G-connected UWB radar system using commercially available hardware and a custom communication module.
- Design and implementation of an IoT platform that supports real-time data visualization, sensor management, and external Application Programming Interface (API) access.
- Experimental validation and performance analysis, including radar accuracy and network latency evaluation in a real-world deployment on a university campus.

The remainder of this paper is structured as follows: Section 2 details the system design, including hardware and software components. Section 3 describes the implementation process, covering both the development of the connected radars and the IoT platform, as well as an initial validation. Section 4 presents the deployment on the university campus. Section 5 analyzes the results, demonstrating the system’s real-time operational capabilities and effectiveness in traffic monitoring. Section 6 discusses the main findings and their implications and outlines potential future research directions. Finally, Section 7 presents the main conclusions of this work.

## 2. Requirements Analysis and System Design

In this section, traffic monitoring requirements are first analyzed to design an end-to-end system capable of satisfying the needs of the UPV campus as an example of a simple urban environment.

### 2.1. Requirements

The requirements for the system have been analyzed and are summarized as follows, all holding equal priority:

- Functionality: The radar system must be capable of detecting vehicles passing through a specific point on a roadway, classifying them, and indicating their speed and direction of movement. Each radar should cover at least two adjacent lanes without an intermediate separation.
- Wireless communication: For efficient, scalable, and seamless data transmission, radars should be able to send their collected data over at least 4G or 5G networks. This connectivity is crucial for enabling real-time data transmission, remote management of the radar units, and integration with a centralized monitoring system.
- Protection: The radar units must be protected against dust and water ingress to ensure they can operate reliably in various environmental conditions. Therefore, the radars should have an IP65 rating.
- Location: The radar units should be able to be conveniently mounted and oriented in various locations, offering flexibility in deployment. The possible installation sites include poles alongside the road, which provide a stable, elevated position for optimal coverage of the monitored lanes, as well as walls or flat surfaces, allowing for flexibility in urban environments where poles may not be feasible.
- Power supply: The radar units should be capable of being powered by batteries, with the option of using solar panels or other renewable energy sources to charge them. Such flexibility enables installation in locations without direct access to the power grid. Additionally, the solution should allow for partial battery recharging during specific times of the day, such as when the electrical lines feeding street lighting are activated. This capability ensures energy efficiency and sustainability, particularly in urban areas where infrastructure may be limited or eco-friendly solutions are prioritized.
- Visualization: The data captured by the mobility radars must be visualized as aggregated time series, considering both the vehicle count (total or by type) and the detected speeds and directions.
- Data access: The platform must allow downloading historical data (CSV and XLS formats will be considered). The data must be available for third parties through a REST API with a clearly defined format.
- Management: The solution must enable the configuration and management of different devices and their activation. The platform must allow multiple-user access. Each user will have access to a specific set of radars. Multiple users can access the data from a radar.
- Deployment and scalability: The platform must support thousands of radar units without significant performance degradation. The platform must be containerized and ready for cloud deployment.
- Connectivity: The web platform must be accessible from the internet, and the connected radars must be able to transmit information using only an internet connection, without requiring dedicated networks.

### 2.2. System Design

The system comprises two main components, whose design is detailed in the following subsections: connected radars and an IoT platform.

#### 2.2.1. Connected Radars

After analyzing Commercial-Off-The-Shelf (COTS) radars, we identified a device from the company uRAD, called uRAD industrial [16], which can be equipped with firmware for vehicle detection and classification. The device is based on the IWR6843AoP chip from Texas Instruments, Dallas, TX, USA, which offers advantages such as high precision, small size, and simplified detection capability. It features integrated antennas, three transmitters, and four receivers. This device has been used previously in other academic works [17,18,19].

The uRAD industrial can work as a Hardware-Attached-on-Top (HAT) of a Raspberry Pi (RPi). The RPi is one of the most popular Single-Board Computers (SBCs) due to its affordability, available interfaces, and extensive community support that facilitates the development of applications. Therefore, RPi has been chosen to control the radar and communication module operations. uRAD provides a reference Python 3.11 script to demonstrate monitoring capabilities, which is run on the RPi while the uRAD industrial uses the detection and classification firmware. The script allows further adaptation to meet the desired solution.

**Figure 1:**
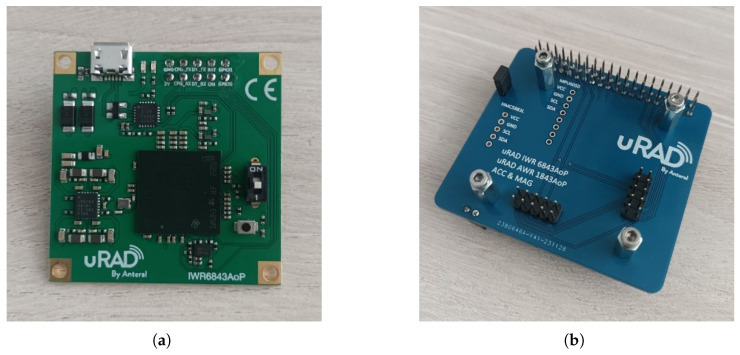
(**a**) uRAD industrial. (**b**) uRAD adapter for RPi.

The radar communicates with the RPi via the UART interface. This connection requires an adapter board, also provided by uRAD, which includes connectors on one side for attaching the uRAD industrial radar, and on the other side, connectors for interfacing with the RPi’s GPIO pins. Figure 1 shows the uRAD radar on the left and the adapter board on the right.

**Figure 2:**
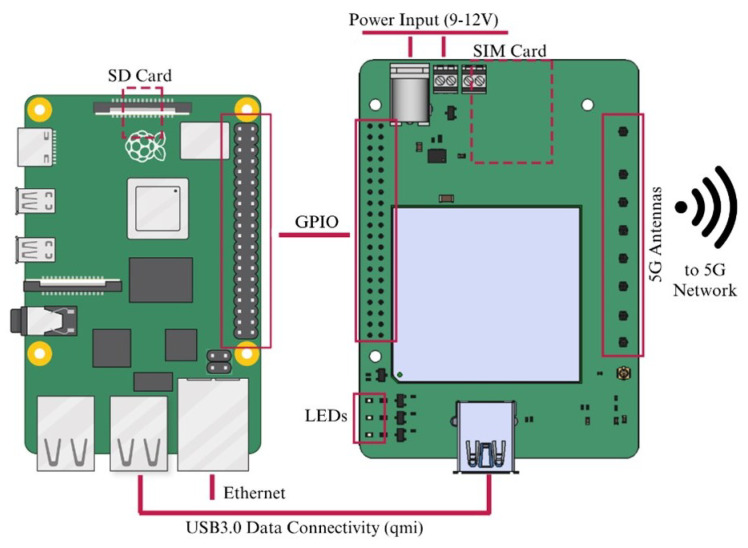
Diagram of the RPi (**left**) + 5GBroad (**right**) combination.

In order to provide cellular communications, a device from Fivecomm, known as 5GBroad, also working as a HAT of an RPi, is included in the design. The 5GBroad board is a communication board based on the RG500Q-EA intelligent broadband module from Quectel, Shanghai, China. It provides connectivity capabilities for 5G SA, NSA, LTE, and 3G networks to the RPi via the USB 3.0 interface. The 5GBroad has been used as a key element to create 5G routers in European research projects, such as in [20]. Additionally, the 5GBroad board serves as the power input for the entire solution, via the GPIO pins, providing a broader input voltage range than the one supported by the RPi. Figure 2 shows the block diagram of the board and its interconnection with the RPi.

**Figure 3:**
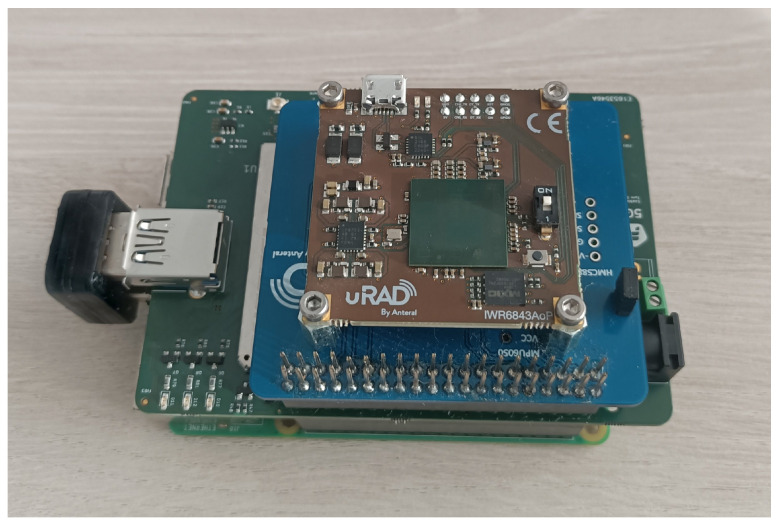
Assembly of radar components.

Figure 3 shows the assembly of the aforementioned components without the enclosure. From top to bottom: the uRAD Industrial module, the uRAD adapter for the RPi, the 5GBroad board, and the Raspberry Pi.

Regarding the enclosure, a commercial box can be adapted to accommodate the necessary hardware.

#### 2.2.2. IoT Platform

The latest advancements in web solutions, database systems, and communication interfaces play a crucial role in designing modern IoT platforms. Trends such as server virtualization, distributed database architectures, and efficient HTTP-based communication interfaces ensure scalability, reliability, and optimal performance.

The IoT platform uses NodeJS for back-end scalability and performance, with an Angular-based front end. The platform also integrates user and permission management and provides API compatibility with external platforms.

The architecture consists of five main components: a Message Queueing Telemetry Transport (MQTT) broker, a proxy, a database, an API, and a web server.

- MQTT Broker: This component receives data from radar devices and filters incoming messages, ensuring authentication and data integrity. RabbitMQ is deployed as the broker, configured for MQTT-based communication.
- Proxy: Acting as an intermediary, the proxy handles communication with the MQTT Broker and stores relevant data in the database. Implemented in NodeJS with TypeScript, it follows SOLID principles for improved maintainability [21].
- Database: The system employs a MySQL relational database deployed on an NAS unit. A RAID 5 configuration enhances data redundancy and storage efficiency, ensuring high-speed read/write operations.
- API: The API serves as the interface between the database and the web platform, handling HTTP requests and optimizing SQL queries. NestJS is used as the framework, ensuring a scalable back-end structure.
- Web Server: The front end is managed via an Angular-based platform, leveraging NodeJS for back-end execution. JSON Web Token (JWT) secure authentication and user access.

**Figure 4:**
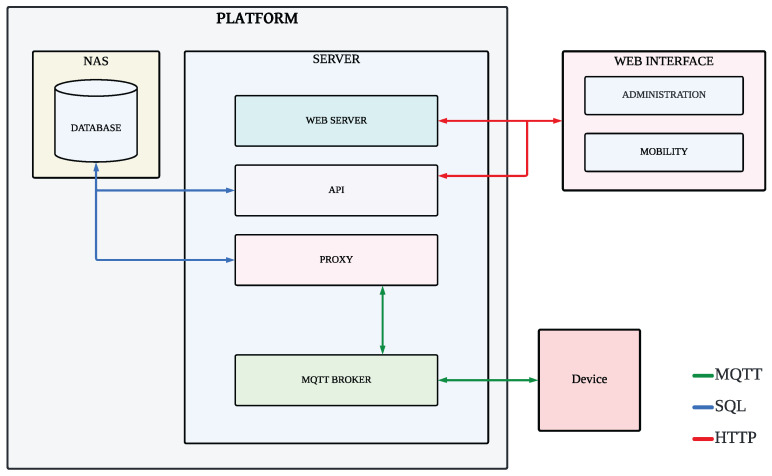
IoT platform design.

This architecture ensures a scalable, secure, and efficient platform for traffic monitoring. Figure 4 illustrates the main components of the platform and the primary data flows between them. Bidirectional arrows indicate two-way communication, with each arrow color representing the underlying technology used: MQTT (green), SQL (blue), and HTTP (red).

## 3. Implementation Details

This section outlines the key aspects of the system’s practical implementation to support its replication. While not exhaustive, the description covers the main relevant topics.

### 3.1. Connected Radars

#### 3.1.1. RaspberryPi 4

For the connected radar solution, two interfaces are essential: the UART interface, used for communication with the radar, and the USB 3.0 interface, required to establish connectivity with the communication board.

Regarding software, the RPi OS Lite was selected as the operating system due to its lightweight nature and lack of a graphical interface, which helps minimize resource usage. Despite its simplicity, it includes several built-in tools useful for system configuration and connectivity management:

- raspi-config: Used to manage device interfaces, including enabling the UART port, configuring remote SSH access, and disabling Wi-Fi and Bluetooth to reduce power consumption and minimize interference.
- raspi-gpio: Enables manual control and configuration of GPIO pin states.
- Python: The programming language used to implement the application that manages radar measurements and transmits data to the MQTT server.
- nmcli and mmcli: Command-line tools used to manage the connectivity of the communication module.

#### 3.1.2. 5GBroad Board

The power-up of the RG500Q-EA module is managed via two GPIO pins. GPIO16 must be set to a high logic level to enable the module’s power supply, while GPIO26 must generate a rising pulse with a duration exceeding 800 ms. A similar pulse can be used subsequently to power down the module.

The module is controlled from the RPi using Qualcomm MSM Interface (QMI) via the mmcli and nmcli tools. Its configuration can also be achieved through AT commands, for which the socat and Minicom tools were installed on the RPi.

#### 3.1.3. uRAD Industrial Radar

The radar includes an adapter board to connect to the RPi. This adapter maps the radar’s interfaces to the RPi’s GPIO pins. The relevant pins are as follows: Pin 8: UART_TX, Pin 10: UART_RX, Pin 29 (GPIO5): Reset, and Pin 31 (GPIO6): ON/OFF.

#### 3.1.4. Electrical Power Solution

Three scenarios were considered for the electrical power supply in urban environments: continuous 24 h power, power supplied by public lighting, and absence of an electrical supply. At the UPV installations, a continuous 24 h supply is available. In this case, only a transformer is required to convert the 220–230 V AC input to the 12 V DC needed to power the system.

#### 3.1.5. Software

The software is based on the Python script provided with the radar board. Additional functionality was added to include MQTT support, along with relevant functions for formatting the message frame to ensure correct decoding by the server. During initial testing, it was observed that the sequential execution of the code handling both radar detections and data transmission to the server introduced a potential issue. Since network connectivity could take longer than initially expected, this sequential processing risked data loss, particularly in high-mobility environments where rapid vehicle detection is crucial.

To mitigate this issue, the execution flow was redesigned to separate radar detection and data transmission into concurrent threads. The main thread now registers vehicle detections into a First-In-First-Out (FIFO) queue using Python’s thread-safe queue module. This change ensures that detections are reliably stored and processed independently of network delays.

**Figure 5:**
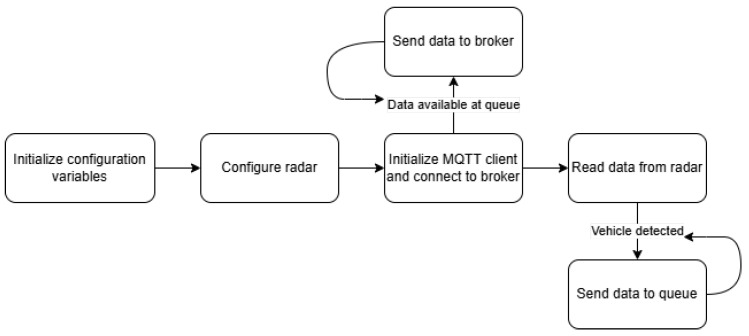
Updated application flowchart with multithreading implementation.

Figure 5 illustrates the flowchart of the solution, integrating the multi-threaded approach.

Further analysis revealed that some radar configuration variables were incorrectly initialized, adversely affecting measurement accuracy. These variables are essential for accurately defining the radar’s placement relative to the roadway. Incorrect values resulted in misinterpretation of the radar output. Table 1 summarizes the corrected configuration parameters. The values provided are illustrative and correspond to one of the radars deployed on the UPV campus.

To improve error diagnosis and debugging capabilities, a logging mechanism was introduced using Python’s built-in logging module. The log files are stored with filenames formatted as <timestamp>_radar.log, allowing developers to track system performance over time.

Finally, an enhancement was made to the function responsible for estimating the lane position of detected vehicles. The original implementation was revised to incorporate two key factors: the lateral displacement of the vehicle and a predefined lane width variable. The inclusion of these variables allows for a more accurate estimation of the vehicle’s position within the lane, thereby enhancing the overall reliability of the lane detection process.

#### 3.1.6. Box and Supports for Machining

**Figure 6:**
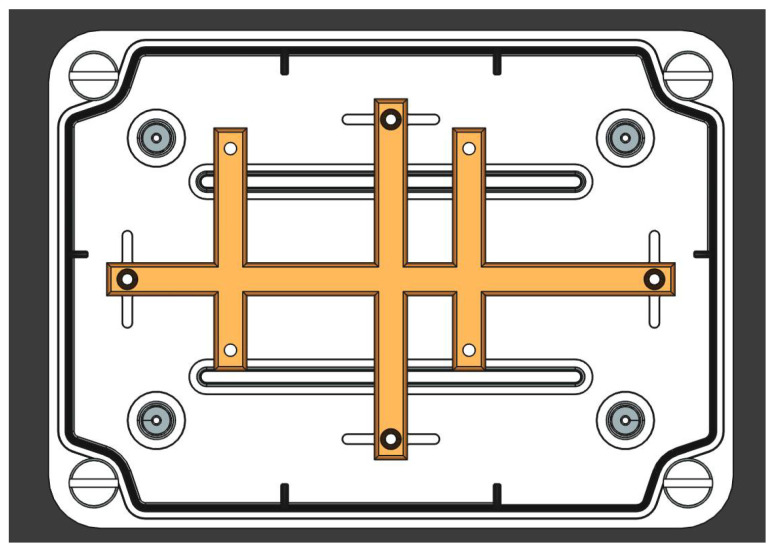
Enclosure and adapter to fix RPi.

For the machining solution, an IP68-rated sealed enclosure was selected. Given that the chosen box was a general-purpose model, it was necessary to fabricate an adapter to modify the internal holes for proper alignment and secure mounting of the hardware. This adapter was produced using resin printing. A top view of the enclosure (white) alongside the adapter piece (yellow) is presented in Figure 6.

**Figure 7:**
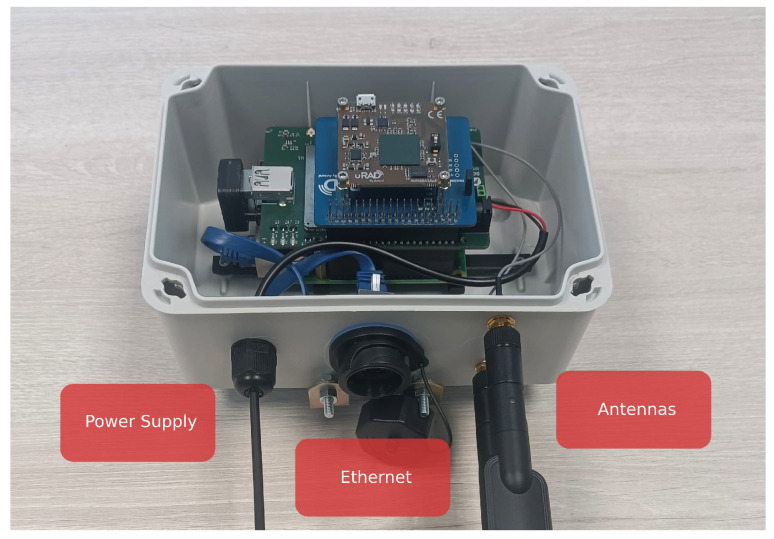
Enclosure with all the components fixed.

The RPi was securely mounted to the adapter piece using two pillars, which provide stability to the 5GBroad board, in addition to two M2.5 screws for further fastening. To facilitate the connection of power cables, antennas, and an Ethernet connector, holes were drilled into the enclosure using a conical drill. This design allows for the integration of components with the RPi without the need to dismantle the final installation. Figure 7 illustrates the enclosure with its lid removed, showcasing the radar, connecting cables, connectors, and antennas contained within.

**Figure 8:**
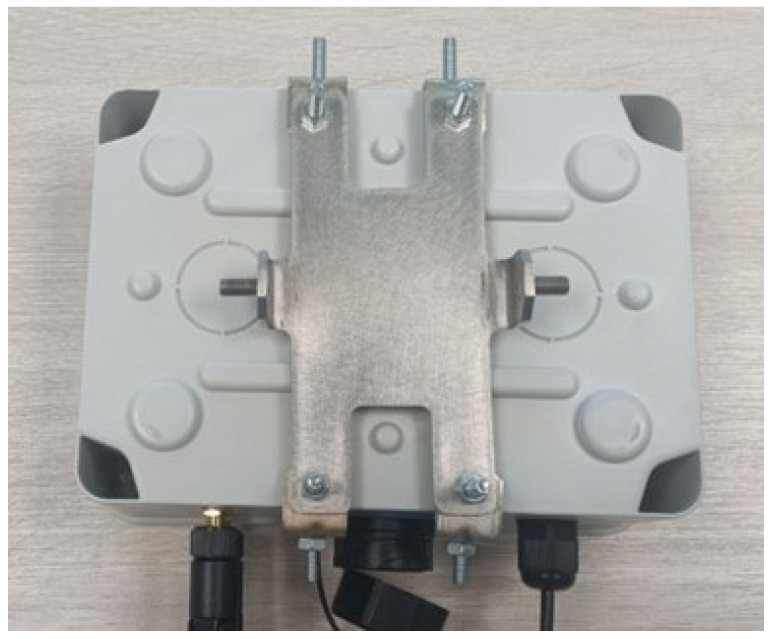
Enclosure’s back with mounting part.

Additional holes were created to implement the mounting solution, allowing for the correct radar orientation in both azimuth and elevation. Figure 8 shows the enclosure’s back with the mounting elements fixed.

### 3.2. IoT Platform

This section provides a detailed description of the steps necessary to replicate the platform, with a focus on the technical implementation of each key component: MQTT broker, proxy, database, API, and web platform. By following these steps, a third party can replicate the entire system accurately.

#### 3.2.1. MQTT Broker

The MQTT broker is deployed using the official RabbitMQ Docker image, with the MQTT protocol explicitly enabled, as it is disabled by default. To make the broker accessible externally, port 8883 must be exposed and traffic must be forwarded from the LAN router to the internal server where the broker is running, allowing radar devices connected via the internet (e.g., through 5G network) to communicate with it. The Docker container should be configured to restart automatically if the server reboots, ensuring continuous operation. Once the container is running, the MQTT plugin needs to be activated by executing the appropriate command within the container, enabling it to handle MQTT messages effectively.

Once the MQTT broker is deployed, security is addressed through device authentication using either X.509 certificates or username/password pairs with SHA-256 or SHA-512 hashing for password encryption. Device-specific credentials are created for secure data transmission. User creation and permission assignment can be automated with commands in the broker container. To secure data transmission, TLS/SSL encryption is enabled by providing RabbitMQ with the server certificate, private key, and CA certificate, ensuring encrypted communication and preventing unauthorized access.

The MQTT broker receives various frames from the radar devices, each with specific configurations. Upon initialization, the device sends initialization frames to the broker at a fixed frequency, including its IMEI as an identifier. If the IMEI is valid, the proxy responds with the radar’s configuration transmitted in JSON format. This configuration encompasses parameters such as user credentials, as well as topics for publishing and subscribing. Once configured, the radar initiates the transmission of measurement data. The transmitted frames contain detailed fields, including vehicle speed, distance to the vehicle, lane, direction, and vehicle type.

#### 3.2.2. Proxy

The proxy component plays a key role in managing the flow of messages between radar devices and the system. Its primary responsibilities include storing received data in the database and handling device configurations and access permissions. Given the need to handle multiple devices simultaneously, optimizing the proxy for efficiency is critical to avoid bottlenecks. To ensure high performance, the proxy is designed to manage high-frequency data transmission through asynchronous processing and optimized database queries, enabling scalability and effective handling of large volumes of data.

NodeJS with TypeScript is used for development due to its non-blocking Input/Output (I/O) model, which supports high concurrency and efficient management of I/O operations. This architecture is particularly well suited for environments with many connected radar devices. TypeScript’s strong typing also improves code maintainability and reduces errors, while the extensive NodeJS ecosystem facilitates seamless integration with other technologies.

The proxy follows an object-oriented programming structure, enhancing scalability and clarity. TypeORM is used for database interaction, allowing efficient mapping between objects and database tables while supporting complex relationships between entities (OneToOne, OneToMany, ManyToMany). This structure ensures both high performance and maintainability as the system expands.

#### 3.2.3. Database Implementation

The MySQL relational database is crucial for storing and managing data transmitted by the radar devices via the MQTT broker, as it not only stores the frames transmitted by the radars but also manages users and supports additional relational logic for accessing information via the web platform. The database setup requires configuring the encoding to utf8mb3 and setting the timezone to UTC. These settings ensure compatibility and consistency across different deployments.

The database is structured into three main groups of tables: device information and configurations (radar, radar_group), user data and device relationships (user_radar, user, user_group), and data transmitted by the radars (frame_daily, fr, frame_rt). The relationships between these tables, such as the many-to-many connection between users and radars through the user_radar table, are designed to support flexible data access and management. The radar table is the central repository for device information, while the user-related tables facilitate access control and permissions for web platform interaction.

The frame tables store radar data and metadata such as timestamps indicating when the data were captured. The frame_rt stores real-time data, while the frame_daily table records daily summaries. Each frame table is linked to the radar table via foreign keys, ensuring that data integrity is maintained. The database schema is carefully designed to handle high-frequency data input, providing fast and efficient access to radar data for analysis and reporting.

#### 3.2.4. API

Once the data are stored in the database, a component is required to retrieve and present it securely and in a standardized way to the user. For this purpose, an API is defined to facilitate request and response messages between the server and the user. The user’s web requests are organized as endpoints. For the implementation of the API, the NestJS framework was selected, as it aligns well with the project’s overarching principles and structure. This choice ensures consistency across components and streamlines integration with the system as a whole. The decision to use NestJS, which is built on top of Node Express JS, was further validated through performance tests that demonstrated its strong capabilities in managing multiple complex queries, making it an optimal solution for this use case.

The directory structure in NestJS follows several established standards, which make it similar to the structure used in the proxy component. Each module in the application is independent and encapsulates its classes, helpers, interfaces, and models. This modular approach promotes reusability and decouples functionality, making it easier to scale and maintain the system. The common directory contains reusable components shared across different modules and introduces new components specifically for the API. These components support implementing various API features, including defining the numerous endpoints required for managing users and radar devices.

The API is structured with a comprehensive set of endpoints to facilitate the management of users and radar devices through the web platform. These endpoints are designed to regulate system access via HTTP requests, providing administrators with the ability to create, delete, and modify records in key database tables, including users, groups, and radars. For users possessing valid JWTs, additional informational endpoints enable access to radar data and related frames, subject to access permissions. Each endpoint is secured using JWT-based authentication, ensuring that only authorized users can retrieve protected resources.

User information is safeguarded through the use of bcrypt hashing for password storage. Bcrypt is a widely adopted algorithm known for its resilience against brute-force attacks, owing to its configurable cost factor, which increases computational complexity as hardware capabilities advance. Furthermore, bcrypt incorporates a unique salt for each password, ensuring that identical plaintext passwords produce distinct hash values. Upon creation by an administrator, a new user does not initially have an active password; the user must first complete a registration process, during which their password is hashed and securely stored. The authentication mechanism subsequently verifies user credentials and enforces access permissions, thereby ensuring secure system access.

#### 3.2.5. Web Platform Implementation with Angular

The solution is designed for integration via the API into either a dedicated web interface or external client web environments. The dedicated interface provides a customized user experience, enabling both end-users and administrators to efficiently view and manage information associated with each device.

Angular has been chosen as the framework for developing the web platform. Angular, a robust open-source framework maintained by Google, offers long-term support and a strong developer community. Its component-based architecture promotes code reusability and enhances the maintainability of the project, making it well suited to the existing API structure. Additionally, Angular’s two-way data binding facilitates seamless synchronization between the user interface and the underlying data model. This means that any change in the User Interface (UI) is automatically reflected in the underlying data model, and vice versa, thereby improving responsiveness and enhancing the overall user experience.

The Angular application adopts a modular architecture in which each module operates independently. This design facilitates the organization of distinct functionalities, enhancing both clarity and scalability. The app’s core directories are housed under the app directory, while graphical and static resources are kept in the assets directory. Key files include the following:

- index.html: contains the main structure for the app’s user interface.
- main.ts: serves as the entry point for executing the application’s logic.
- style.css: defines the global styles for the application.

Each Angular module follows a consistent structure, incorporating components, routes, and services that are dedicated to specific features. This modular and well-organized structure not only supports scalability and maintainability but also leverages Angular’s core capabilities to enable the development of a high-performance, dynamic, and responsive web platform.

#### 3.2.6. Visual Design of the Web Platform

The visual design of the web platform plays a crucial role in ensuring that the user experience extends beyond aesthetics. A well-designed interface enhances functionality, accessibility, and intuitiveness. Thoughtful design improves navigation, allowing users to easily access and interpret the necessary information, an essential feature in complex web applications where interaction with multiple data types and interfaces is required.

The platform offers three main scenarios, each with two distinct layouts. These scenarios include the homepage, the map, and the dashboard, with an additional exclusive administration page. The design ensures that the layout is consistent across all pages, with some pages, like the authentication ones, having a slightly different layout for specific functions.

**Figure 9:**
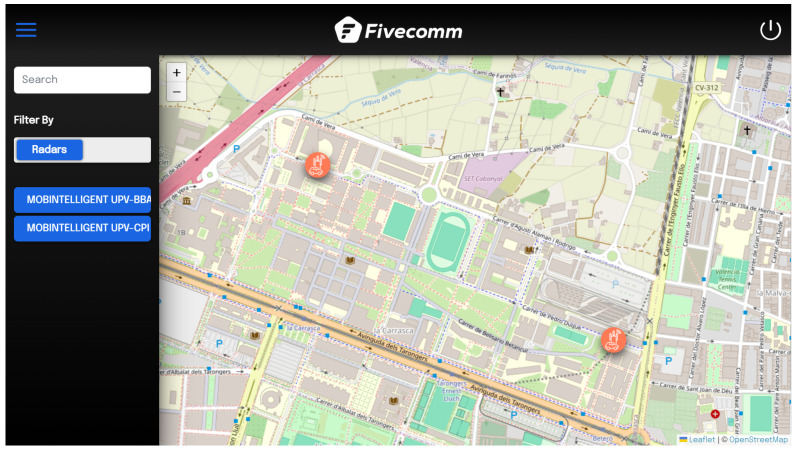
Web user interface: map view.

Once a user successfully authenticates and logs into the platform, the first page they encounter is the map view. The map shows the locations of devices, and a left-side dropdown menu allows users to filter or search for specific devices. A fixed top menu, which remains constant across other pages except the authentication pages, provides quick access to essential navigation options. Figure 9 shows the map interface with the side menu expanded.

The side menu in the map view enables users to filter by devices or device groups and search for specific devices by name. This makes it easy to locate any radar or device on the map. When a user clicks on a radar, a preview window appears, providing the option to access the specific dashboard for that device. This window displays key information and provides a quick gateway to more detailed views.

**Figure 10:**
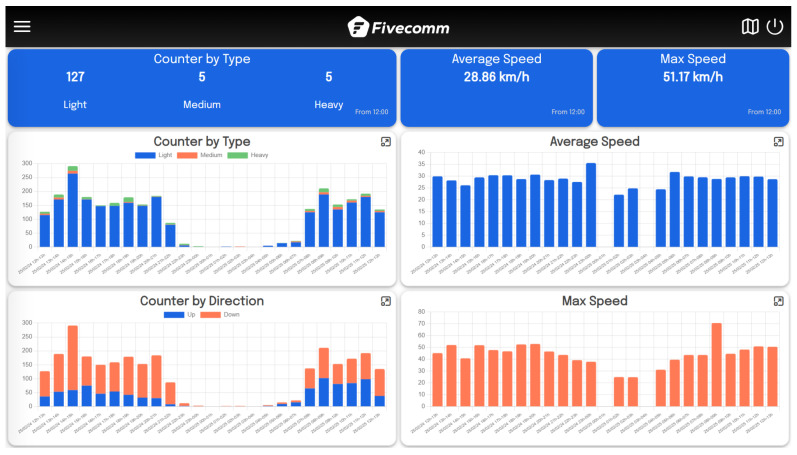
Web user interface: dashboard view.

After selecting a device, the user is redirected to a detailed dashboard. The platform supports different types of dashboards, including mobility and level-based radars. For instance, when accessing a mobility radar dashboard, the user is presented with four interactive graphs, which include readings for the radar by type, direction, and both maximum and average speeds. The top three cards also display the latest data for quick reference, helping users quickly interpret the most relevant information. Users can customize their data view by adjusting the type of frames, granularity, and interval from the dropdown menu on the left. Figure 10 shows an example of the dashboard.

In addition to the user-facing features, an administration page has been developed to manage both devices and users. This page allows administrators to configure and control various platform aspects, ensuring that the system operates smoothly and securely. The admin page is designed to be intuitive, providing all the necessary tools in one place.

Several libraries were utilized to streamline the development of the web interface and enhance its functionality. First, Leaflet is used to integrate the interactive map, allowing users to visualize and interact with device locations easily. Second, ChartJS is implemented to create dynamic and responsive graphs, displaying key metrics like speed and radar type. Finally, PrimeNG is a comprehensive library used to add ready-to-use UI components and styles, helping speed up development while maintaining a clean and modern look. The combination of these libraries with Angular’s powerful features ensures that the platform is both visually appealing and functionally robust. The overall design, layout, and use of these libraries help create an engaging, intuitive, and responsive web experience for users and administrators.

#### 3.2.7. Technology Stack

As a summary, to replicate the system, the following technologies are essential:

- RabbitMQ with MQTT plugin: for communication between radar devices and the proxy.
- Node.js and NestJS: For back-end services, including API creation and handling radar data.
- MySQL: for storing radar- and user-related data in a relational database.
- Angular: for creating the user interface with a focus on reusability and modularity.
- JWT: for secure authentication of users.
- bcrypt: for securely hashing user passwords during authentication.

### 3.3. Functional Validation: Laboratory Environment over 4G/5G Networks

A validation was conducted in a laboratory environment at the Fivecomm office, covering the entire system to ensure all components functioned as they would in an operational environment.

An initial evaluation tested whether object detection triggered the correct transmission of information to the server.

A metallic plate was moved directly over the radar to simulate an object. After a detection, the system correctly generated the data frame, queued it for processing via the MQTT thread, and successfully published it. Each message was printed in a console window, and a visual inspection confirmed that the delay from object movement to message display was consistently under one second.

**Figure 11:**
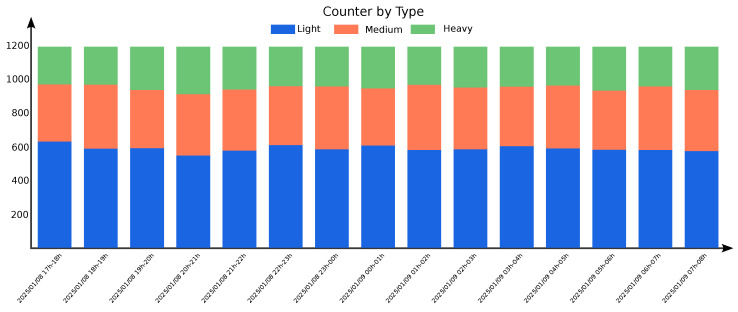
Test results showing message transmission every 3 s.

To validate the communications component, messages with random parameters were generated every three seconds. A random distribution was used, with half of the messages representing light vehicles, one-third medium vehicles, and one-sixth heavy vehicles. This test assessed the stability of server connectivity, verifying the reception of 1200 messages per hour during the test run, confirming that no packet loss occurred during the test run. Figure 11 shows the results of this 15 h test, where each bar represents the number of messages received in one-hour intervals. As shown, each bar reaches the expected count of 1200 messages, and the distribution of vehicle types matches the specified proportions.

## 4. Deployment

### 4.1. Radars

The installation of radars on the UPV campus is preceded by a preliminary study phase to determine the most suitable locations. These locations are selected based on three main criteria: their relevance for UPV in terms of the information that can be gathered, the feasibility of installation considering the physical characteristics of the available sites and power supply availability, and the assurance that the radars will function correctly under real operating conditions.

Concerning this latter criterion, several factors can significantly impact radar performance and must be carefully considered. One critical factor is the presence of frequent stops and starts of vehicle movement within the detection area (e.g., in congested zones or intersections). Each acceleration event can introduce false vehicle detections, affecting data accuracy. Similarly, very low speeds may cause misclassifications, leading to the false detection of large vehicles instead of lighter ones. Another key aspect is the precise alignment of the radar towards the detection area. Proper orientation is essential to maximize detection accuracy and minimize blind spots. The azimuth and elevation angles were determined through a combination of manufacturer recommendations and on-site calibration trials. Potential sources of interference were also analyzed, leading to the avoidance of installations near large reflective surfaces, such as metallic structures or parked vehicles, that could introduce signal degradation.

Based on these criteria, an initial survey was conducted across multiple candidate sites on the UPV campus. For each location, installation feasibility was evaluated by assessing available mounting surfaces, power access points, line-of-sight to the road, and potential interference sources. Following this, short-term radar deployments were carried out using portable tripods and powered by portable batteries at shortlisted locations. These temporary setups enabled the collection of preliminary detection data, which were used to identify potential issues such as signal reflections, misalignments, or coverage gaps. The calibration process involved fine-tuning the azimuth and elevation angles of the radar units through iterative adjustments, guided by both real-time detection feedback and comparison with ground-truth observations. Only after confirming consistent performance under realistic traffic conditions were the radar units permanently installed. This structured selection and calibration methodology ensured that each installation site maximized radar effectiveness while minimizing data noise and detection errors.

Two locations were finally selected for radar installation, based on the outcomes of the site surveys and calibration tests. The first location (Location 1) is a two-lane road with one lane per direction, situated in the western part of the campus near the Fine Arts Faculty. This site offered good visibility and mounting options, and the calibration tests confirmed stable operation under real traffic conditions. The radar is oriented towards the inner part of the campus. In contrast, the opposite direction was discarded to avoid interference from a nearby pedestrian crossing, where frequent stop-and-start movements could compromise detection accuracy. The second location (Location 2) is a single-lane road near an exit on the eastern side of the campus, close to the City of Innovation. It was selected for its strategic value in monitoring outbound traffic and for offering a low-interference environment, which supported reliable performance in the calibration phase. Here, the radar is also directed towards the inner part of the campus to optimize data collection.

**Figure 12:**
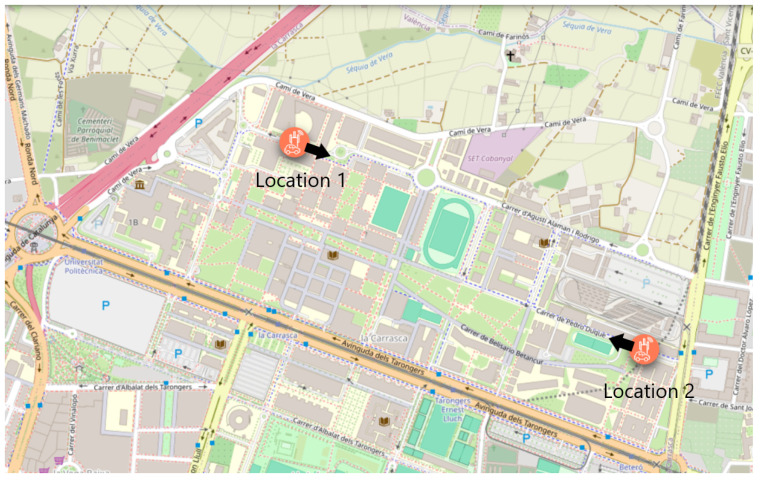
Map of the campus area with the location and orientation of the connected radars.

**Figure 13:**
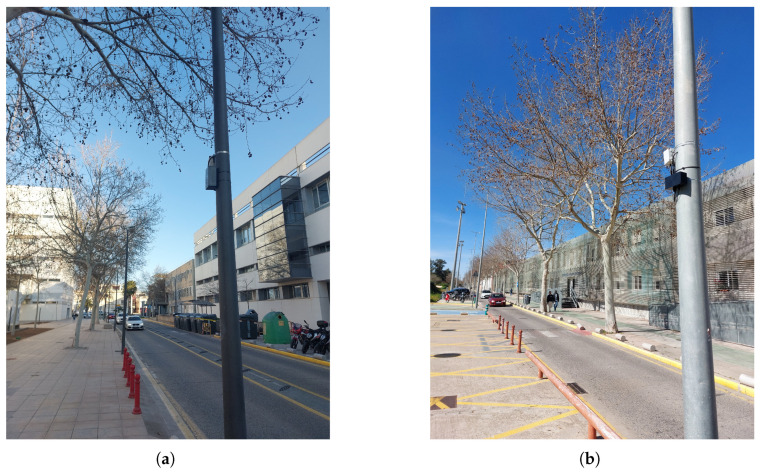
Detection areas for the radars: (**a**) Location 1 and (**b**) Location 2.

Figure 12 shows a map of the campus with the locations of the two radars installed. The black arrows indicate the direction of the radar pointing. Figure 13 shows the detection areas of the radars and their surroundings.

**Figure 14:**
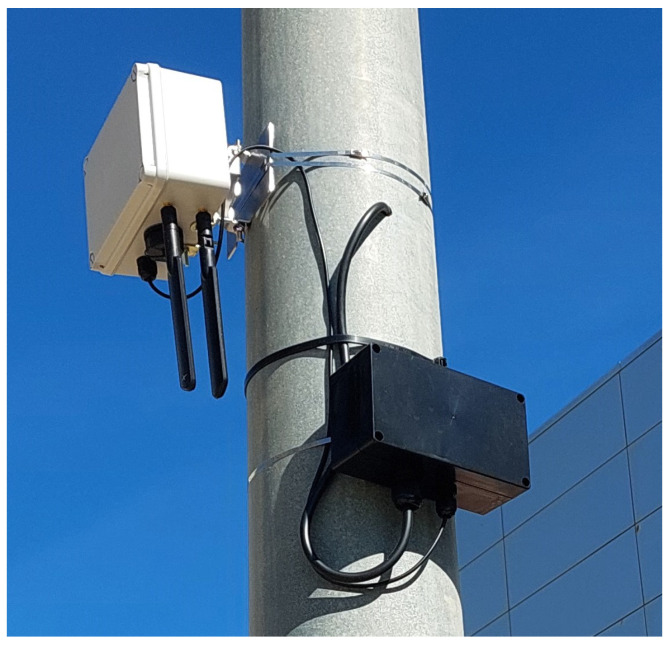
Radar installation detail showing the two enclosures.

In both locations, the radar was mounted on a pole with a 230 V power supply. In addition to the radar housing described in the machining section, a secondary enclosure was installed to house a 230 V to 12 V, 3 A power adapter. Figure 14 shows the installation detail with both enclosures.

The radar mounting mechanism allowed precise orientation adjustments. According to uRAD specifications, the azimuth angle, defined as the angle between the radar’s pointing direction and the line parallel to the closest lane, is set to 15º at Location 1 and 0º at Location 2. In both cases, the radar is tilted at an elevation angle of 11º relative to the horizontal, with a mounting height of 3 m.

### 4.2. IoT Platform

For the deployment of the solution, having a straightforward procedure is crucial to ensure an efficient and error-free application launch. The first step is to configure the server where the application will be hosted, installing the required software such as NodeJs, RabbitMQ, NestJS, and the corresponding libraries and dependencies. Afterward, the application needs to be built, including the proxy, API, and web interface, all developed in TypeScript, requiring a transpilation process to JavaScript.

However, to simplify this process, Docker images can be created that encapsulate the entire configuration, allowing faster and more consistent deployment. Docker containers were chosen over traditional virtual machines due to their lightweight nature, easy replication, and rapid scalability. The use of Docker ensures a more modular and easily maintainable system, reducing deployment complexity and enabling seamless updates in future iterations. Official images are used for the MQTT broker and MySQL database. In contrast, custom images are created for the web server, API, and proxy, each containing all necessary libraries, dependencies, and environment variables for configuration. The proxy image uses an official Node-Alpine image, optimized for size. The process begins with a Node base image, to which the distribution directory is transferred, resulting in a final size of 180 MB. The API image follows a similar process, with adjustments to environment variables (such as timezone and port exposure) and has a final size of 242 MB. The web interface image also follows an optimized structure, with compilation in a separate stage, resulting in a final size of 150 MB.

## 5. Results

This section presents a set of results of the traffic monitoring conducted at both locations for several weeks, as visualized through the IoT platform dashboard, and an experiment to evaluate the accuracy of the system results and the communication latency.

### 5.1. Traffic Monitoring Results

#### 5.1.1. Location 1

**Figure 15:**
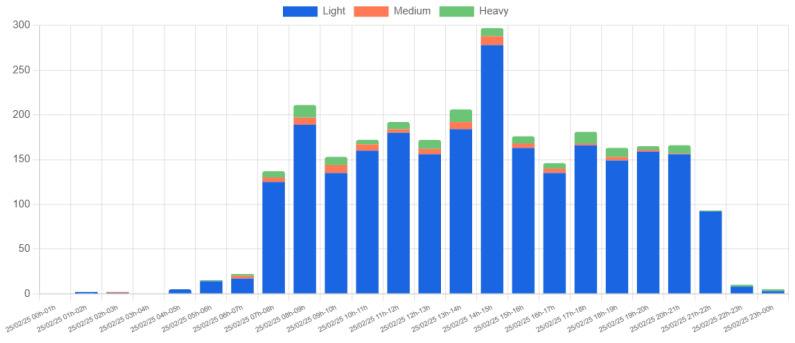
Hourly vehicle count by type at Location 1 on 25 February 2025.

Figure 15 shows the hourly vehicle count for a single day (25 February 2025) at Location 1. The detected vehicles are categorized into three types: light, medium, and heavy, based on their size. The data reveal a predominance of light vehicles, with two significant peaks occurring between 08:00–09:00 and 14:00–15:00. These peaks align with the typical university schedule, reflecting the arrival and departure of students and staff. The higher peak in the 14:00–15:00 interval is likely due to overlapping entry and exit movements.

**Figure 16:**
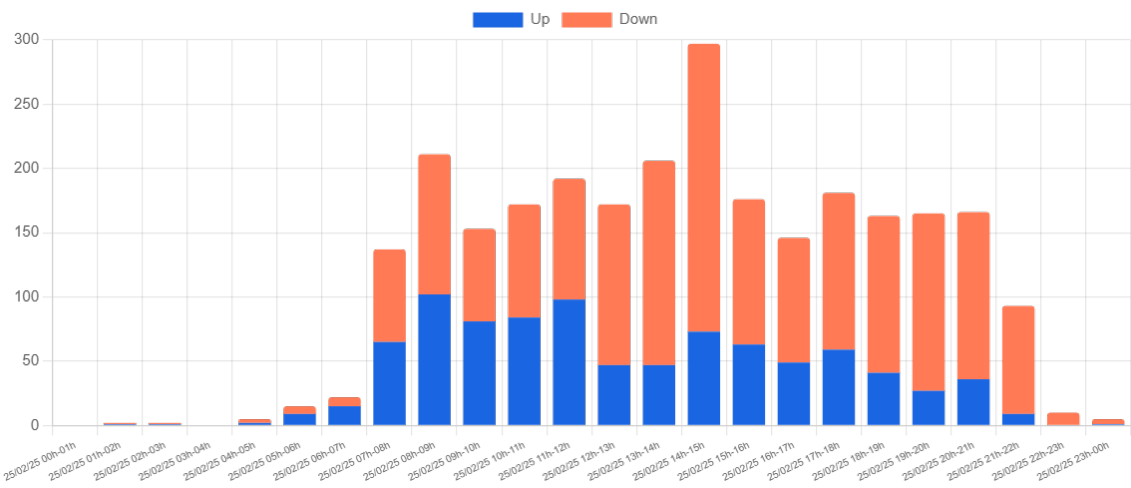
Hourly vehicle count by direction at Location 1 on 25 February 2025.

Figure 16 provides additional insight by showing the distribution of vehicle movement directions. The data indicate that the highest volume of arrivals occurs between 08:00–09:00, while departures peak between 13:00–15:00. These observations are consistent with expected traffic patterns on a university campus, and no anomalies or irregularities were detected.

#### 5.1.2. Location 2

**Figure 17:**
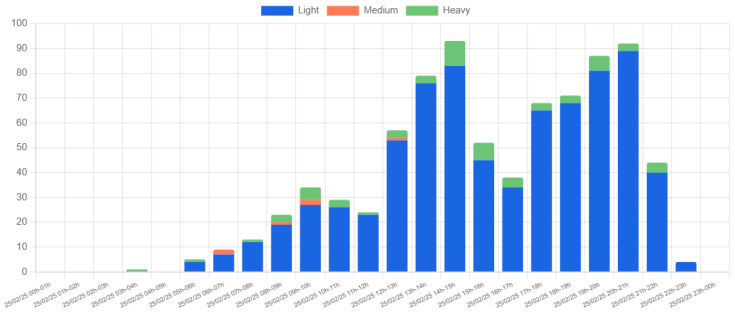
Hourly vehicle count by type at Location 2 on 25 February 2025.

Figure 17 illustrates the vehicle count at Location 2 during the same day. Compared to Location 1, the most pronounced peaks occur between 14:00–15:00 and 20:00–21:00. This shift is expected, given that Location 2 is situated near a campus exit, where outbound traffic is more prominent later in the day. Since this radar is installed on a single-lane road, the count by direction has been omitted.

#### 5.1.3. Weekly and Multi-Week Trends

**Figure 18:**
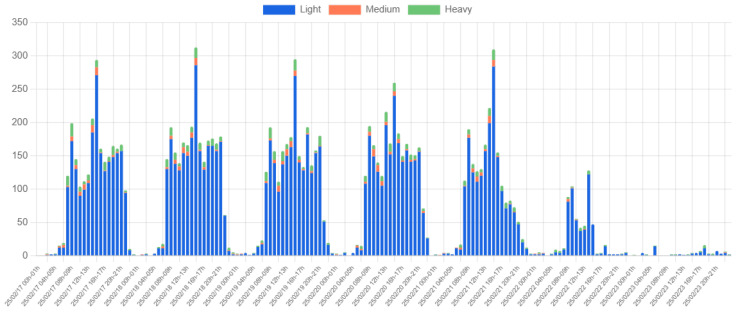
Weekly vehicle count by type at Location 1.

Figure 18 presents vehicle count data collected over a whole week to analyze longer-term traffic patterns. A consistent traffic pattern emerges from Monday to Friday, with peak intervals resembling those observed in the single-day data. However, traffic volume decreases significantly on Friday afternoon, aligning with the university’s flexible work hours. Some vehicle activity is observed on Saturday morning, but the remainder of the weekend shows minimal traffic, likely limited to security personnel movement.

**Figure 19:**
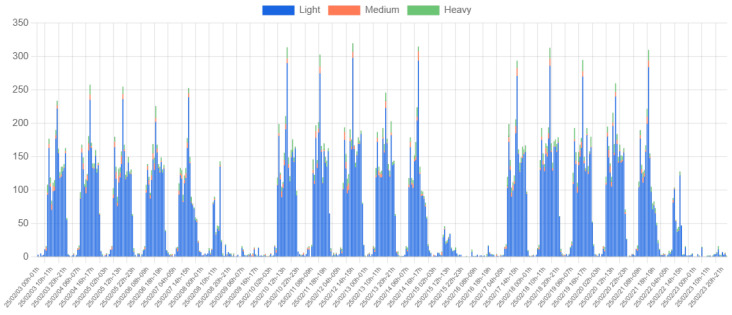
Three-week vehicle count by type at Location 1.

Expanding the analysis further, Figure 19 presents data collected over three weeks. The cyclic nature of campus traffic is evident, with weekly patterns repeating across the dataset. Notably, the first week exhibits slightly lower traffic volumes, likely due to reduced activity following the end-of-term examinations before regular classes resumed.

#### 5.1.4. Speed Analysis

**Figure 20:**
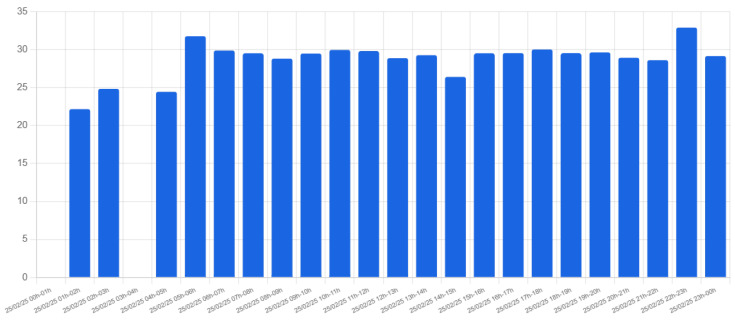
Average speed of vehicles at Location 1 on 25 February 2025.

**Figure 21:**
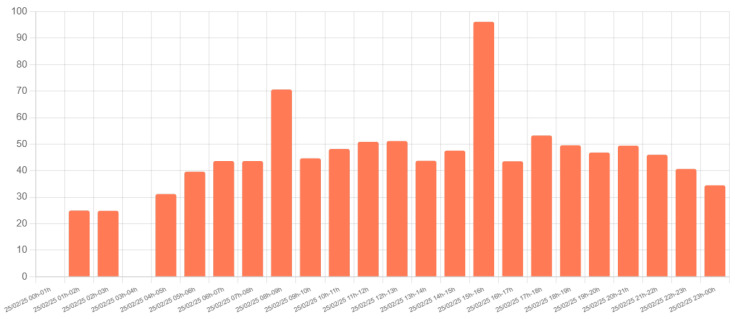
Maximum speed of vehicles at Location 1 on 25 February 2025.

Figure 20 and Figure 21 display the average and maximum speeds recorded at Location 1 on 25 February. The absence of bars in some intervals indicates periods with no detected traffic. The data highlight instances of high speeds, which could warrant intervention by university administrators to address potential safety concerns.

### 5.2. Evaluation of Detection Accuracy and Latency

To validate the accuracy and reliability of the proposed radar-based traffic monitoring system, an experiment was conducted at one of the radar deployment locations during peak traffic hours. The objective was to compare the system’s detected vehicle counts, classifications, and direction estimations with manually recorded observations. The communication latency was also measured to evaluate the system’s real-time operation.

#### 5.2.1. Experiment Setup

During one hour, the busy hour from 13:00 to 14:00, a video recording was taken in the detection area of the radar at Location 1, allowing for the manual recording of the number of passing vehicles, their classifications, including bicycles, scooters, motorbikes, light cars, vans, and trucks, and their travel direction. The recorded data were then compared with the output of the traffic monitoring platform.

#### 5.2.2. Results and Analysis

The comparative analysis between the observations and the radar system’s detections is summarized in Table 2.

The system achieved a detection rate of 94.81%, representing the proportion of detected vehicles relative to the total observed. Most missing detections (13 out of 16) occurred when closely spaced vehicles were detected as a single entity. The false detection rate (also known in the literature as false alarm rate) remained low at 1.02%, demonstrating the robustness of the system in filtering out erroneous detections. Although acceptable thresholds can vary depending on the application, in non-critical traffic monitoring contexts such as this one, where the data inform planning rather than immediate control decisions, a false detection rate below 5% is generally considered acceptable. Therefore, the observed performance is deemed suitable for the intended use case.

A missed detection in traffic monitoring could result in underestimating traffic volume and delaying necessary interventions, such as adjusting signal timings or providing real-time traffic guidance. On the other hand, a false detection may cause unnecessary system responses, such as traffic light changes or the activation of congestion alerts, which could lead to inefficient traffic flow and increased operational costs. Both detection issues can impact the accuracy of traffic models, thereby affecting long-term planning decisions and real-time traffic management. However, it is important to note that these errors may compensate for each other when focusing on the total number of vehicles circulating. Specifically, the missed detections, which represent a small fraction (only 16 out of 308 vehicles observed, or about 5%), can be offset by false alarms, which are even lower in number (3 out of 295 total detections, or about 1%). As a result, the overall impact on the total traffic volume estimate is expected to be very low, leading to a more balanced and reliable traffic count.

In terms of classification performance, the system achieved a classification accuracy of 97.29%, assuming that motorbikes were grouped with light vehicles. A total of eight misclassifications were observed, with five instances resulting from closely spaced vehicles being misinterpreted as a single medium-sized vehicle rather than multiple light vehicles. The direction accuracy was notably high at 99.66%, with only one instance of incorrect direction estimation.

To assess the communication latency in the developed traffic monitoring system, we measured, for each detection, the time elapsed from the moment the radar transmits an MQTT packet to the server until it receives a response from the server. This Round Trip Time (RTT) measurement provides insight into the end-to-end latency introduced by the 5G network and system processing. Since RTT accounts for both uplink and downlink transmission delays, the one-way latency can be approximated as half of the RTT. Given the real-time nature of traffic monitoring, maintaining a low and consistent latency is crucial for ensuring timely and accurate vehicle detection and classification.

**Figure 22:**
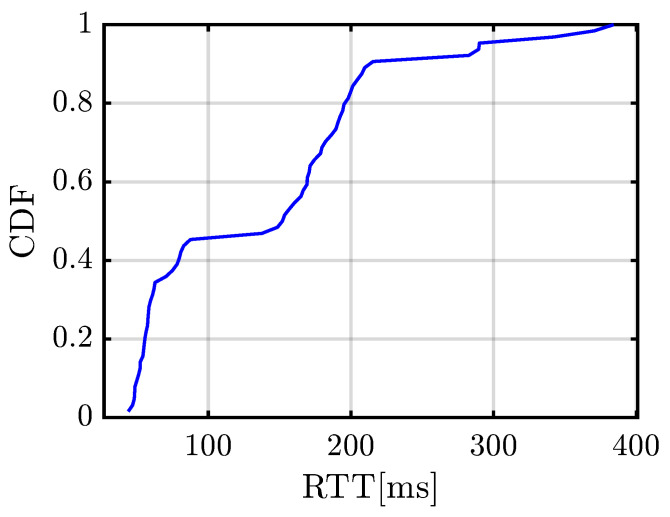
CDF of the measured RTT values defined as the time elapsed from the moment the radar transmits an MQTT packet to the server until it receives a response from the server.

The collected RTT values range from approximately 43.9 ms to 384.1 ms, reflecting the variability in network conditions and processing times. The empirical cumulative distribution function (Cumulative Distribution Function (CDF)) of the RTT, shown in Figure 22, provides a clearer understanding of latency distribution.

Key observations from the analysis include that the median RTT is around 150 ms, indicating that at least half of the transmissions experience a delay below this threshold. The maximum observed RTT of 384.1 ms represents an upper bound under the tested conditions. Although such delays could introduce minor inconsistencies in time-sensitive applications, this is not the case for the application under study. The lowest recorded RTT of 43.9 ms demonstrates that the system is capable of operating with minimal latency in optimal conditions. A significant portion of RTT values cluster below 200 ms, suggesting that the system maintains a stable latency in most cases. Given an RTT median of 150 ms, the estimated one-way latency is 75 ms, which is well within the acceptable range for real-time traffic monitoring applications. Note that we consider an interaction with a latency below 100 ms to be perceived by a human as an instantaneous interaction [22].

The RTT measurements confirm that the developed system achieves low-latency operation over a commercial 5G network, with most RTT values well below 200 ms. This level of performance ensures near-real-time data transmission, which is critical for accurate vehicle detection and classification.

## 6. Discussion

As presented in the Results Section, our system achieves a classification accuracy of approximately 93% and a detection rate above 95%, which are consistent with the performance reported in the literature across various sensing technologies. Camera-based systems have demonstrated classification accuracies above 95% under favorable conditions [8]. At the same time, inductive loop detectors typically achieve vehicle count accuracies between 92% and 99% [4,8], although their classification capabilities are more limited. Similarly, [6] reports detection rates above 98% and classification accuracies exceeding 96% using a commercial radar module, and [13] reports up to 95% accuracy in structured urban environments using a 77 GHz FMCW radar. These results validate that our solution, despite being based on a compact, low-cost, and easily deployable architecture, delivers competitive performance compared to established alternatives, supporting its feasibility for scalable urban traffic monitoring applications.

It is important to clarify that a quantitative comparative analysis with other solutions (such as image-based surveillance or loop detectors) is beyond the scope of this study. Our objective is not to demonstrate superiority in terms of raw detection or classification performance. As noted in the Introduction, camera- and loop-based systems have shown high levels of accuracy. Instead, this work emphasizes that a radar-based solution can achieve comparable detection and classification performance while offering distinct advantages in deployment flexibility, energy efficiency, privacy, and cost.

Notably, our solution also offers a competitive cost and energy consumption profile. The proposed system is built entirely with COTS components, resulting in an approximate unit cost of EUR 600. Moreover, it operates with a power consumption below 5.5 W, making it suitable for energy-constrained or solar-powered deployments. These characteristics support the feasibility of large-scale installations, particularly in scenarios where low operational costs and energy efficiency are critical.

Installation complexity is another key consideration. The proposed radar-based platform requires only basic pole or roadside mounting and minimal alignment, significantly reducing deployment time and cost.

Data privacy concerns are substantially mitigated in our solution. Unlike video systems that may capture identifiable information such as license plates or faces, radar sensors only detect objects’ physical motion and presence. Using this information significantly reduces privacy risks and supports compliance with regulations such as the GDPR, making radar-based monitoring preferable in privacy-sensitive contexts.

Scalability is a central feature of the proposed solution. Regarding server dimensioning for the MQTT broker, each active connection consumes approximately 30 KB of RAM. Benchmark results for high-performance, memory-optimized virtual machines indicate that a single CPU core can process roughly 1,500 MQTT messages per second under typical load conditions. These numbers reflect the low computational requirements of the system and its potential for scalable deployment. Furthermore, given that radar frame size is only 52 bytes, even in high-traffic situations involving around 200 vehicles per 5 min [23], the average network load remains minimal (approximately 35 Bps).

Future work will focus on optimizing the system for real-world deployment. In particular, improving message handling and server-side processing is crucial to minimize occasional high RTT values, thereby ensuring consistent real-time performance. The platform will also be evaluated under different network loads and environmental conditions (such as weather, RF interference, and urban congestion) to assess its robustness and reliability outside the controlled campus environment. For city-scale deployment, further developments may include expanding sensor coverage, integrating machine learning for predictive traffic analysis, and implementing real-time feedback mechanisms (e.g., dynamic speed regulation or adaptive traffic light control). Additionally, the integration of complementary sensor types (e.g., cameras, acoustic sensors) will be explored to assess whether sensor fusion can enhance detection accuracy and adaptability in complex urban scenarios.

## 7. Conclusions

This study demonstrated the feasibility and performance of a 5G-connected UWB radar-based traffic monitoring platform deployed on a university campus. The key conclusions are as follows:

- Full-system scope: This work presents an end-to-end radar-based monitoring solution (covering sensing, 5G communication, cloud integration, and data visualization) built entirely using COTS components.
- Real-world validation: The system was validated through deployment in a campus environment. Unlike prior works that focus narrowly on radar hardware or lab simulations, our work contributes a full-stack, deployable IoT solution for smart mobility applications.
- Cost and deployment feasibility: The use of commercially available components (UWB radar, Raspberry Pi, custom 5G module, and enclosure) results in a unit cost of approximately EUR 600. With a power consumption under 5.5 W, the system is suitable for energy-efficient or solar-powered installations. Minimal alignment and mounting requirements enable rapid, large-scale deployment.
- Scalability and network efficiency: The MQTT-based IoT platform supports high scalability. Radar frames of just 52 bytes and efficient server-side message handling ensure low computational and bandwidth demands, making the solution viable for large-scale urban deployments.
- Privacy and compliance: Unlike video-based systems, radar sensors capture no personally identifiable information, reducing privacy risks and ensuring compliance with regulations such as the GDPR. This makes the system particularly suitable for privacy-sensitive scenarios.
- Performance evaluation: Field testing on a double-lane road during peak traffic hours demonstrated robust performance: 94.81% detection rate, 1.02% false detection rate, 97.29% classification accuracy, and 99.66% direction accuracy. The median end-to-end latency over a commercial 5G network was 75 ms. These results validate the system’s capability to deliver accurate and real-time traffic monitoring, even under challenging conditions and non-dedicated networks.
- Implications for smart campus and urban mobility: The system provided insights into vehicle flow and speed variations. The collected data confirmed expected traffic patterns, such as peak congestion during morning and afternoon hours, and revealed occasional excessive speed. These insights support data-driven traffic management strategies, including entry/exit control, dynamic parking policies, and pedestrian safety enhancements. The findings contribute to the broader vision of intelligent transportation systems and smart campus development.

## Figures and Tables

**Table 1:** Radar configuration variables.

Variable	Value	Description
USB_COMMUNICATION	False	Defines whether communication is via USB
USB_CONNECTOR_UPWARD	True	Indicates radar USB connector orientation
pitch_angle	15°	Radar tilt angle relative to vertical
yaw_angle	15°	Radar angle relative to road axis
VELOCITY_POSITIVE	True	Detects vehicles approaching the radar
VELOCITY_NEGATIVE	True	Detects vehicles moving away from the radar

**Table 2:** Performance evaluation of the radar detection system.

Metric	Symbol/Expression	Value	Description
Total vehicles observed	$N_{\mathrm{obs}}$	308	Manually recorded vehicle count
Total detections	$N_{\det}$	295	Vehicles detected by the radar system
Missing detections	$N_{\mathrm{miss}}$	16	Vehicles not detected by the system
False detections	$N_{\mathrm{false}}$	3	Incorrect vehicle detections by the system
Correct detections	$N_{\mathrm{correct}}$	292	Correct vehicle detections by the system
Detection rate (%)	$\frac{N_{\det}}{N_{\mathrm{obs}}}\times 100$	94.81	Percentage of detected vehicles relative to total observed
False detection rate (%)	$\frac{N_{\mathrm{false}}}{N_{\det}}\times 100$	1.02	Percentage of incorrect detections relative to total detected
Misclassified vehicles	$N_{\mathrm{misclass}}$	8	Number of vehicles incorrectly classified
Incorrect direction estimations	$N_{\mathrm{dir\_wrong}}$	1	Number of vehicles with wrong movement direction
Classification accuracy (%)	$\frac{N_{\det}-N_{\mathrm{misclass}}}{N_{\det}}\times 100$	97.29	Percentage of correctly classified vehicles
Direction accuracy (%)	$\frac{N_{\det}-N_{\mathrm{dir\_wrong}}}{N_{\det}}\times 100$	99.66	Percentage of correct direction estimations

## Data Availability

Data are contained within the article.
